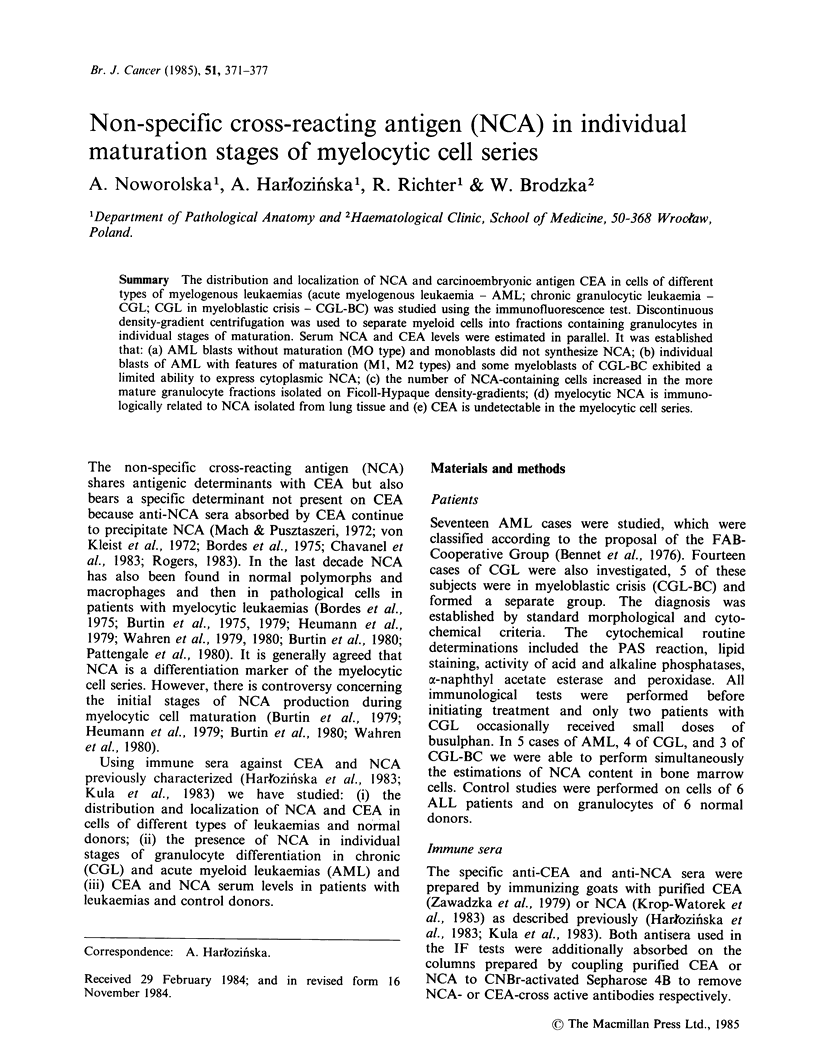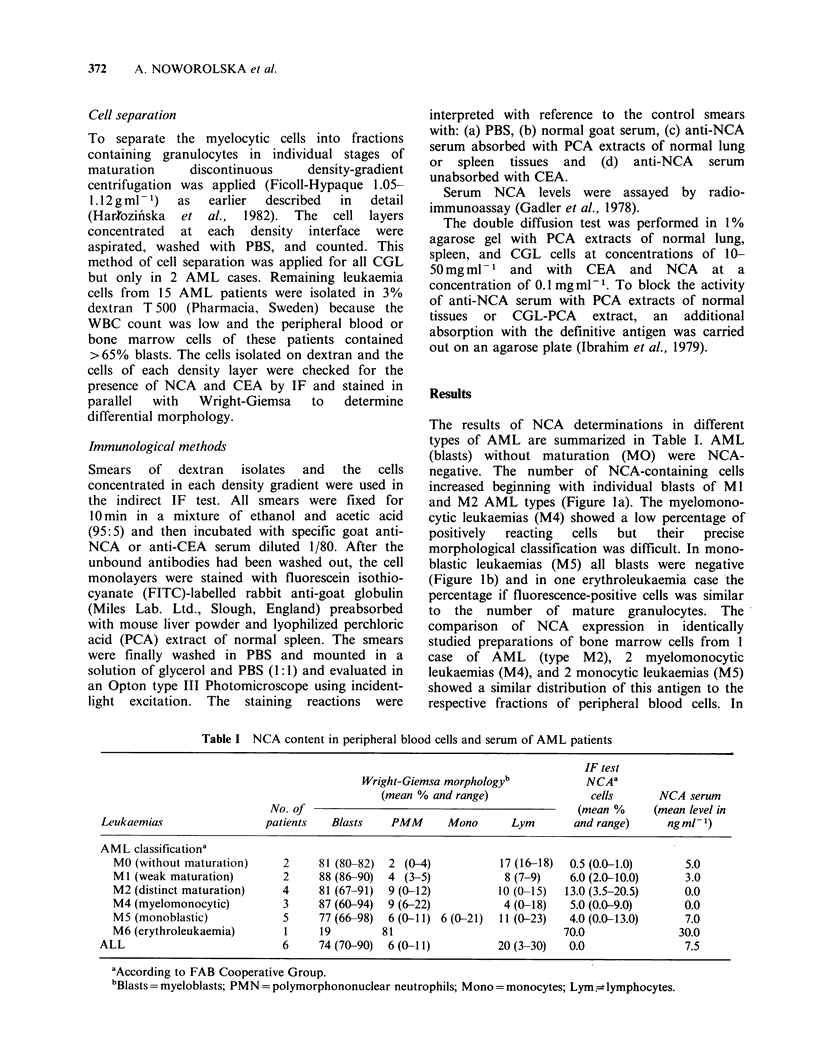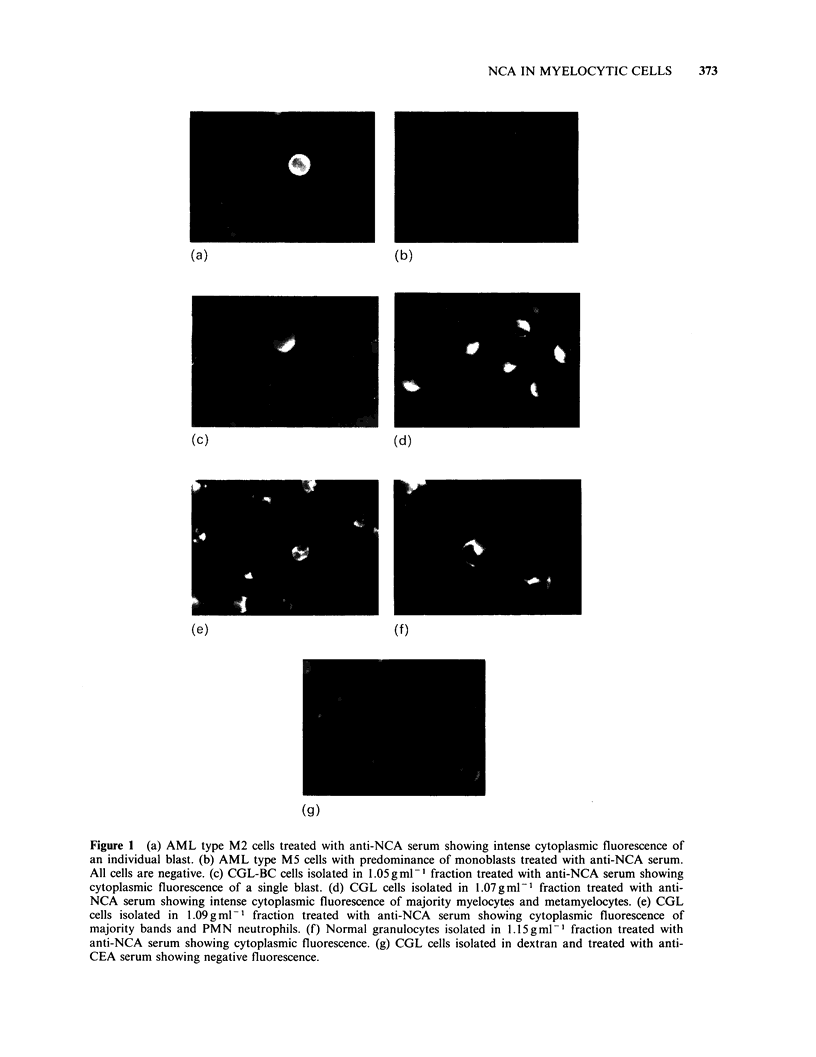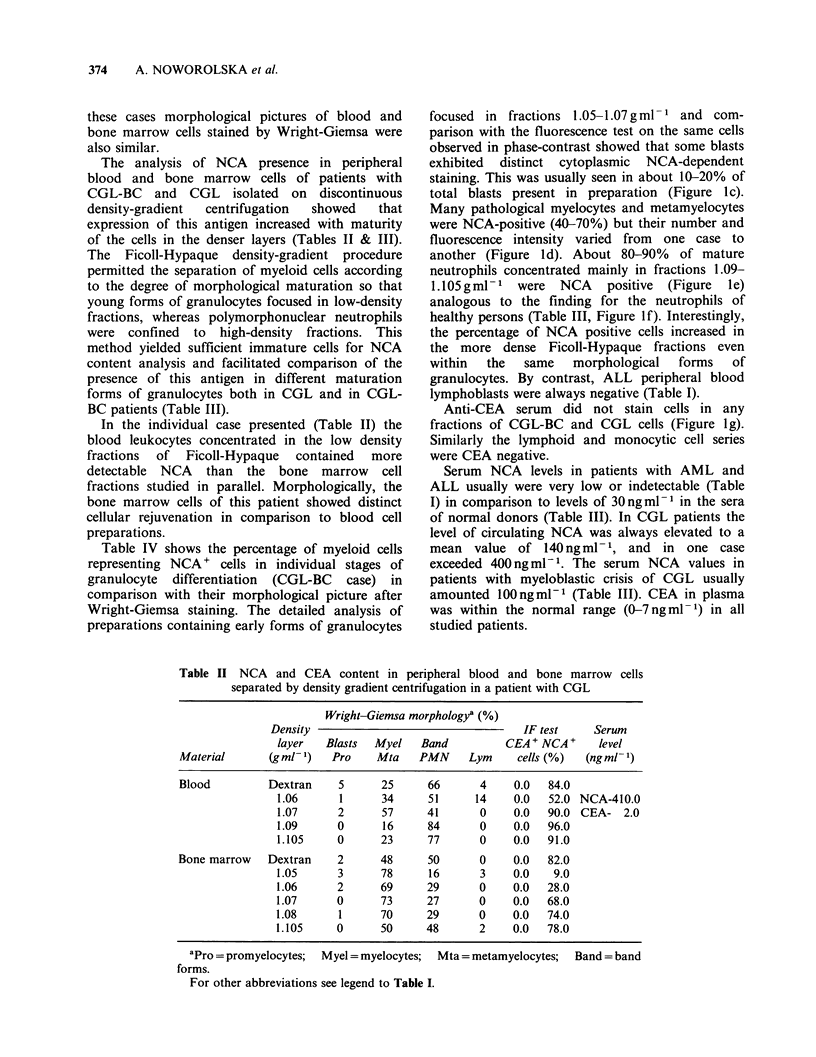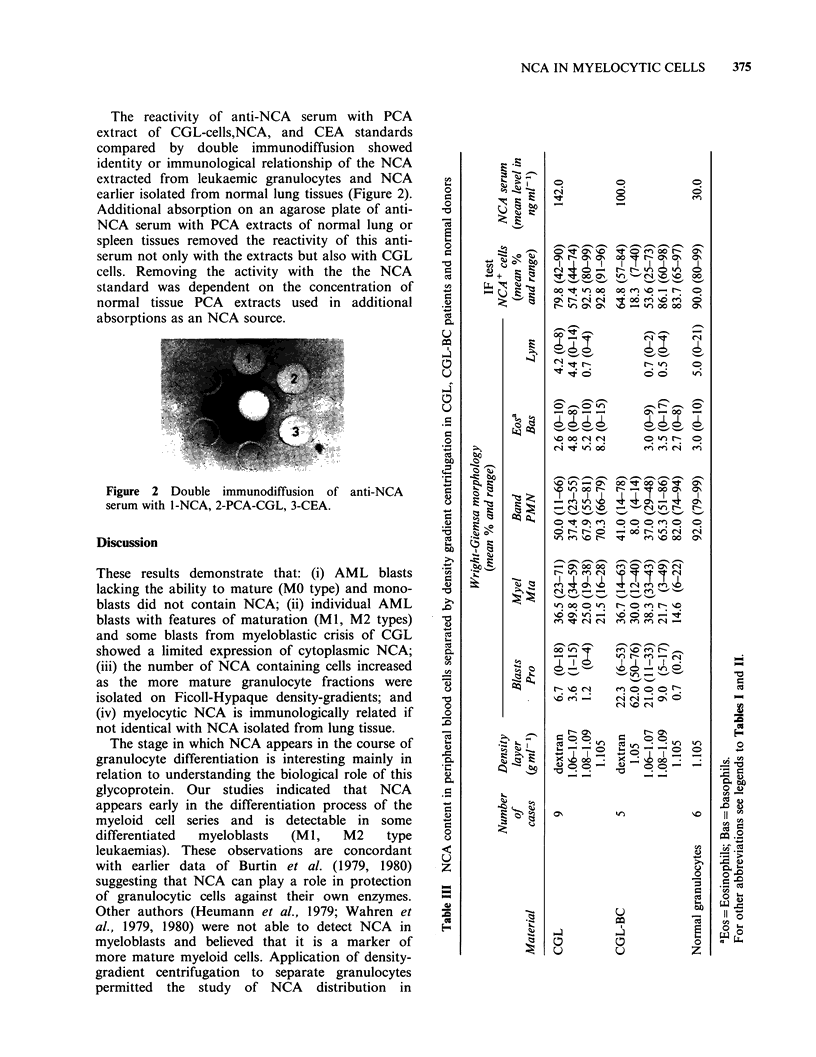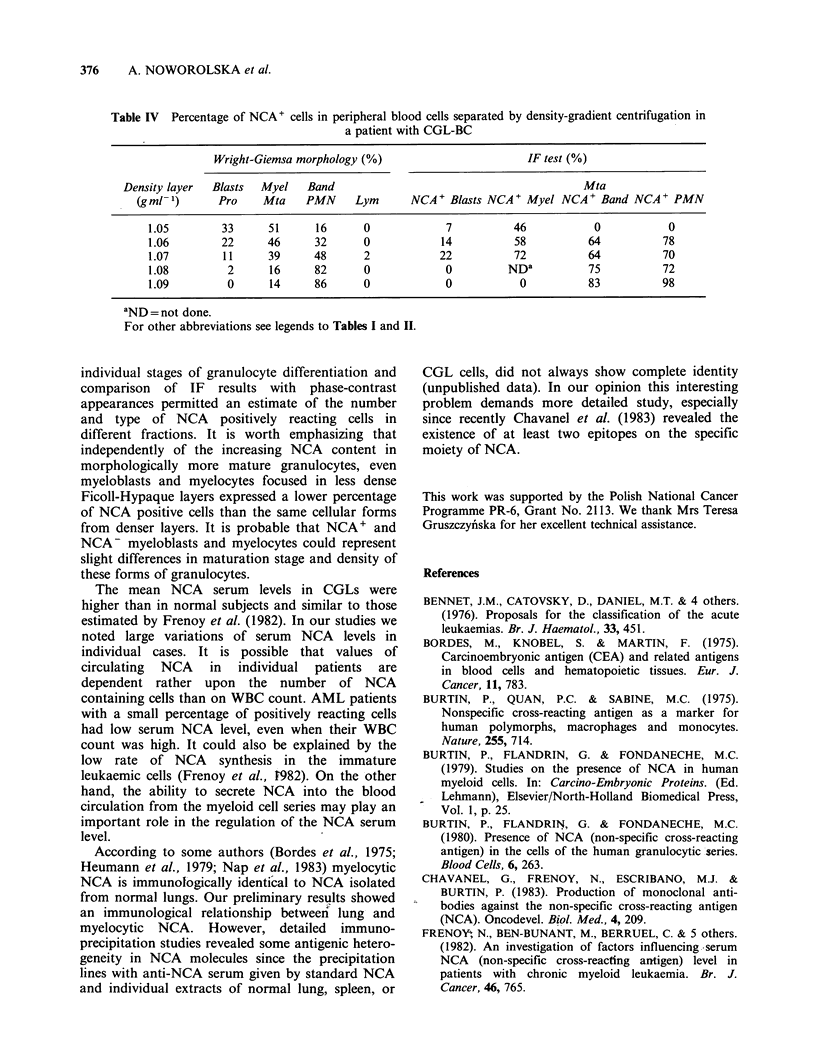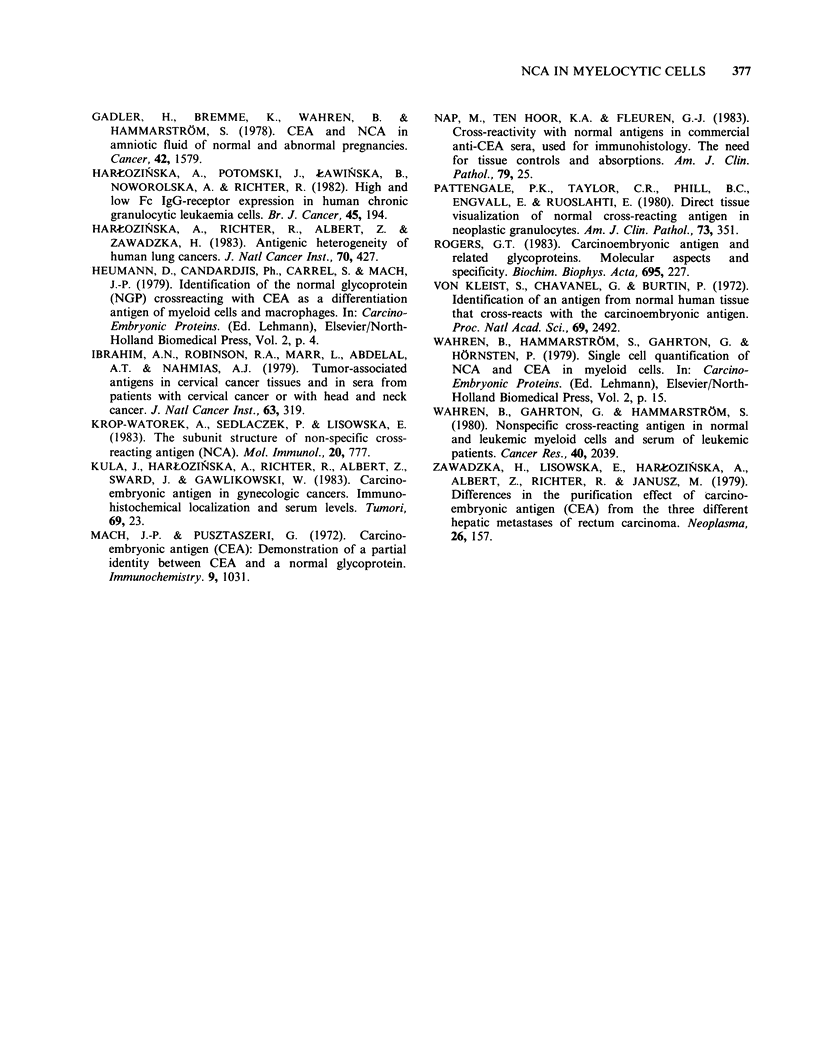# Non-specific cross-reacting antigen (NCA) in individual maturation stages of myelocytic cell series.

**DOI:** 10.1038/bjc.1985.49

**Published:** 1985-03

**Authors:** A. Noworolska, A. Harłozińska, R. Richter, W. Brodzka

## Abstract

**Images:**


Br. J. Cancer (1985), **51**, 371–377

### Non-specific cross-reacting antigen (NCA) in individual maturation stages of myelocytic cell series

A. Noworolska[1], A. Harłozińska[1], R. Richter[1] & W. Brodzka[2]

[1]*Department of Pathological Anatomy and* [2]*Haematological Clinic, School of Medicine, 50-368 Wrocław, Poland.*

**Summary** The distribution and localization of NCA and carcinoembryonic antigen CEA in cells of different types of myelogenous leukaemias (acute myelogenous leukaemia – AML; chronic granulocytic leukaemia – CGL; CGL in myeloblastic crisis – CGL-BC) was studied using the immunofluorescence test. Discontinuous density-gradient centrifugation was used to separate myeloid cells into fractions containing granulocytes in individual stages of maturation. Serum NCA and CEA levels were estimated in parallel. It was established that: (a) AML blasts without maturation (MO type) and monoblasts did not synthesize NCA; (b) individual blasts of AML with features of maturation (M1, M2 types) and some myeloblasts of CGL-BC exhibited a limited ability to express cytoplasmic NCA; (c) the number of NCA-containing cells increased in the more mature granulocyte fractions isolated on Ficoll-Hypaque density-gradients; (d) myelocytic NCA is immunologically related to NCA isolated from lung tissue and (e) CEA is undetectable in the myelocytic cell series.

The non-specific cross-reacting antigen (NCA) shares antigenic determinants with CEA but also bears a specific determinant not present on CEA because anti-NCA sera absorbed by CEA continue to precipitate NCA (Mach & Pusztaszeri, 1972; von Kleist *et al.*, 1972; Bordes *et al.*, 1975; Chavanel *et al.*, 1983; Rogers, 1983). In the last decade NCA has also been found in normal polymorphs and macrophages and then in pathological cells in patients with myelocytic leukaemias (Bordes *et al.*, 1975; Burtin *et al.*, 1975, 1979; Heumann *et al.*, 1979; Wahren *et al.*, 1979, 1980; Burtin *et al.*, 1980; Pattengale *et al.*, 1980). It is generally agreed that NCA is a differentiation marker of the myelocytic cell series. However, there is controversy concerning the initial stages of NCA production during myelocytic cell maturation (Burtin *et al.*, 1979; Heumann *et al.*, 1979; Burtin *et al.*, 1980; Wahren *et al.*, 1980).

Using immune sera against CEA and NCA previously characterized (Harłozińska *et al.*, 1983; Kula *et al.*, 1983) we have studied: (i) the distribution and localization of NCA and CEA in cells of different types of leukaemias and normal donors; (ii) the presence of NCA in individual stages of granulocyte differentiation in chronic (CGL) and acute myeloid leukaemias (AML) and (iii) CEA and NCA serum levels in patients with leukaemias and control donors.

Correspondence: A. Harłozińska.

Received 29 February 1984; and in revised form 16 November 1984.

### Materials and methods

### *Patients*

Seventeen AML cases were studied, which were classified according to the proposal of the FAB-Cooperative Group (Bennet *et al.*, 1976). Fourteen cases of CGL were also investigated, 5 of these subjects were in myeloblastic crisis (CGL-BC) and formed a separate group. The diagnosis was established by standard morphological and cytochemical criteria. The cytochemical routine determinations included the PAS reaction, lipid staining, activity of acid and alkaline phosphatases, α-naphthyl acetate esterase and peroxidase. All immunological tests were performed before initiating treatment and only two patients with CGL occasionally received small doses of busulphan. In 5 cases of AML, 4 of CGL, and 3 of CGL-BC we were able to perform simultaneously the estimations of NCA content in bone marrow cells. Control studies were performed on cells of 6 ALL patients and on granulocytes of 6 normal donors.

### *Immune sera*

The specific anti-CEA and anti-NCA sera were prepared by immunizing goats with purified CEA (Zawadzka *et al.*, 1979) or NCA (Krop-Watorek *et al.*, 1983) as described previously (Harłozińska *et al.*, 1983; Kula *et al.*, 1983). Both antisera used in the IF tests were additionally absorbed on the columns prepared by coupling purified CEA or NCA to CNBr-activated Sepharose 4B to remove NCA- or CEA-cross active antibodies respectively.

© The Macmillan Press Ltd., 1985

*Cell separation*

To separate the myelocytic cells into fractions containing granulocytes in individual stages of maturation discontinuous density-gradient centrifugation was applied (Ficoll-Hypaque 1.05–1.12 g ml$^{-1}$) as earlier described in detail (Harłozińska *et al.*, 1982). The cell layers concentrated at each density interface were aspirated, washed with PBS, and counted. This method of cell separation was applied for all CGL but only in 2 AML cases. Remaining leukaemia cells from 15 AML patients were isolated in 3% dextran T 500 (Pharmacia, Sweden) because the WBC count was low and the peripheral blood or bone marrow cells of these patients contained >65% blasts. The cells isolated on dextran and the cells of each density layer were checked for the presence of NCA and CEA by IF and stained in parallel with Wright-Giemsa to determine differential morphology.

*Immunological methods*

Smears of dextran isolates and the cells concentrated in each density gradient were used in the indirect IF test. All smears were fixed for 10 min in a mixture of ethanol and acetic acid (95:5) and then incubated with specific goat anti-NCA or anti-CEA serum diluted 1/80. After the unbound antibodies had been washed out, the cell monolayers were stained with fluorescein isothiocyanate (FITC)-labelled rabbit anti-goat globulin (Miles Lab. Ltd., Slough, England) preabsorbed with mouse liver powder and lyophilized perchloric acid (PCA) extract of normal spleen. The smears were finally washed in PBS and mounted in a solution of glycerol and PBS (1:1) and evaluated in an Opton type III Photomicroscope using incident-light excitation. The staining reactions were interpreted with reference to the control smears with: (a) PBS, (b) normal goat serum, (c) anti-NCA serum absorbed with PCA extracts of normal lung or spleen tissues and (d) anti-NCA serum unabsorbed with CEA.

Serum NCA levels were assayed by radioimmunoassay (Gadler *et al.*, 1978).

The double diffusion test was performed in 1% agarose gel with PCA extracts of normal lung, spleen, and CGL cells at concentrations of 10–50 mg ml$^{-1}$ and with CEA and NCA at a concentration of 0.1 mg ml$^{-1}$. To block the activity of anti-NCA serum with PCA extracts of normal tissues or CGL-PCA extract, an additional absorption with the definitive antigen was carried out on an agarose plate (Ibrahim *et al.*, 1979).

### Results

The results of NCA determinations in different types of AML are summarized in Table I. AML (blasts) without maturation (MO) were NCA-negative. The number of NCA-containing cells increased beginning with individual blasts of M1 and M2 AML types (Figure 1a). The myelomonocytic leukaemias (M4) showed a low percentage of positively reacting cells but their precise morphological classification was difficult. In monoblastic leukaemias (M5) all blasts were negative (Figure 1b) and in one erythroleukaemia case the percentage if fluorescence-positive cells was similar to the number of mature granulocytes. The comparison of NCA expression in identically studied preparations of bone marrow cells from 1 case of AML (type M2), 2 myelomonocytic leukaemias (M4), and 2 monocytic leukaemias (M5) showed a similar distribution of this antigen to the respective fractions of peripheral blood cells. In

**Table I** NCA content in peripheral blood cells and serum of AML patients

| *Leukaemias* | *No. of patients* | *Wright-Giemsa morphology*[b] (*mean % and range*) | | | | *IF test NCA*[a] *cells* (*mean % and range*) | *NCA serum* (*mean level in ng ml*$^{-1}$) |
|---|---|---|---|---|---|---|---|
| | | *Blasts* | *PMM* | *Mono* | *Lym* | | |
| AML classification[a] | | | | | | | |
| M0 (without maturation) | 2 | 81 (80–82) | 2 (0–4) | | 17 (16–18) | 0.5 (0.0–1.0) | 5.0 |
| M1 (weak maturation) | 2 | 88 (86–90) | 4 (3–5) | | 8 (7–9) | 6.0 (2.0–10.0) | 3.0 |
| M2 (distinct maturation) | 4 | 81 (67–91) | 9 (0–12) | | 10 (0–15) | 13.0 (3.5–20.5) | 0.0 |
| M4 (myelomonocytic) | 3 | 87 (60–94) | 9 (6–22) | | 4 (0–18) | 5.0 (0.0–9.0) | 0.0 |
| M5 (monoblastic) | 5 | 77 (66–98) | 6 (0–11) | 6 (0–21) | 11 (0–23) | 4.0 (0.0–13.0) | 7.0 |
| M6 (erythroleukaemia) | 1 | 19 | 81 | | | 70.0 | 30.0 |
| ALL | 6 | 74 (70–90) | 6 (0–11) | | 20 (3–30) | 0.0 | 7.5 |

[a]According to FAB Cooperative Group.
[b]Blasts = myeloblasts; PMN = polymorphononuclear neutrophils; Mono = monocytes; Lym = lymphocytes.

**Figure 1** (a) AML type M2 cells treated with anti-NCA serum showing intense cytoplasmic fluorescence of an individual blast. (b) AML type M5 cells with predominance of monoblasts treated with anti-NCA serum. All cells are negative. (c) CGL-BC cells isolated in $1.05\,g\,ml^{-1}$ fraction treated with anti-NCA serum showing cytoplasmic fluorescence of a single blast. (d) CGL cells isolated in $1.07\,g\,ml^{-1}$ fraction treated with anti-NCA serum showing intense cytoplasmic fluorescence of majority myelocytes and metamyelocytes. (e) CGL cells isolated in $1.09\,g\,ml^{-1}$ fraction treated with anti-NCA serum showing cytoplasmic fluorescence of majority bands and PMN neutrophils. (f) Normal granulocytes isolated in $1.15\,g\,ml^{-1}$ fraction treated with anti-NCA serum showing cytoplasmic fluorescence. (g) CGL cells isolated in dextran and treated with anti-CEA serum showing negative fluorescence.

these cases morphological pictures of blood and bone marrow cells stained by Wright-Giemsa were also similar.

The analysis of NCA presence in peripheral blood and bone marrow cells of patients with CGL-BC and CGL isolated on discontinuous density-gradient centrifugation showed that expression of this antigen increased with maturity of the cells in the denser layers (Tables II & III). The Ficoll-Hypaque density-gradient procedure permitted the separation of myeloid cells according to the degree of morphological maturation so that young forms of granulocytes focused in low-density fractions, whereas polymorphonuclear neutrophils were confined to high-density fractions. This method yielded sufficient immature cells for NCA content analysis and facilitated comparison of the presence of this antigen in different maturation forms of granulocytes both in CGL and in CGL-BC patients (Table III).

In the individual case presented (Table II) the blood leukocytes concentrated in the low density fractions of Ficoll-Hypaque contained more detectable NCA than the bone marrow cell fractions studied in parallel. Morphologically, the bone marrow cells of this patient showed distinct cellular rejuvenation in comparison to blood cell preparations.

Table IV shows the percentage of myeloid cells representing $NCA^+$ cells in individual stages of granulocyte differentiation (CGL-BC case) in comparison with their morphological picture after Wright-Giemsa staining. The detailed analysis of preparations containing early forms of granulocytes focused in fractions 1.05–1.07 $g\,ml^{-1}$ and comparison with the fluorescence test on the same cells observed in phase-contrast showed that some blasts exhibited distinct cytoplasmic NCA-dependent staining. This was usually seen in about 10–20% of total blasts present in preparation (Figure 1c). Many pathological myelocytes and metamyelocytes were NCA-positive (40–70%) but their number and fluorescence intensity varied from one case to another (Figure 1d). About 80–90% of mature neutrophils concentrated mainly in fractions 1.09–1.105 $g\,ml^{-1}$ were NCA positive (Figure 1e) analogous to the finding for the neutrophils of healthy persons (Table III, Figure 1f). Interestingly, the percentage of NCA positive cells increased in the more dense Ficoll-Hypaque fractions even within the same morphological forms of granulocytes. By contrast, ALL peripheral blood lymphoblasts were always negative (Table I).

Anti-CEA serum did not stain cells in any fractions of CGL-BC and CGL cells (Figure 1g). Similarly the lymphoid and monocytic cell series were CEA negative.

Serum NCA levels in patients with AML and ALL usually were very low or indetectable (Table I) in comparison to levels of 30 $ng\,ml^{-1}$ in the sera of normal donors (Table III). In CGL patients the level of circulating NCA was always elevated to a mean value of 140 $ng\,ml^{-1}$, and in one case exceeded 400 $ng\,ml^{-1}$. The serum NCA values in patients with myeloblastic crisis of CGL usually amounted 100 $ng\,ml^{-1}$ (Table III). CEA in plasma was within the normal range (0–7 $ng\,ml^{-1}$) in all studied patients.

**Table II** NCA and CEA content in peripheral blood and bone marrow cells separated by density gradient centrifugation in a patient with CGL

| *Material* | *Density layer* ($g\,ml^{-1}$) | *Wright–Giemsa morphology*[a] (%) *Blasts Pro* | *Myel Mta* | *Band PMN* | *Lym* | *IF test cells* (%) $CEA^+$ | $NCA^+$ | *Serum level* ($ng\,ml^{-1}$) |
|---|---|---|---|---|---|---|---|---|
| Blood | Dextran | 5 | 25 | 66 | 4 | 0.0 | 84.0 | |
| | 1.06 | 1 | 34 | 51 | 14 | 0.0 | 52.0 | NCA-410.0 |
| | 1.07 | 2 | 57 | 41 | 0 | 0.0 | 90.0 | CEA- 2.0 |
| | 1.09 | 0 | 16 | 84 | 0 | 0.0 | 96.0 | |
| | 1.105 | 0 | 23 | 77 | 0 | 0.0 | 91.0 | |
| Bone marrow | Dextran | 2 | 48 | 50 | 0 | 0.0 | 82.0 | |
| | 1.05 | 3 | 78 | 16 | 3 | 0.0 | 9.0 | |
| | 1.06 | 2 | 69 | 29 | 0 | 0.0 | 28.0 | |
| | 1.07 | 0 | 73 | 27 | 0 | 0.0 | 68.0 | |
| | 1.08 | 1 | 70 | 29 | 0 | 0.0 | 74.0 | |
| | 1.105 | 0 | 50 | 48 | 2 | 0.0 | 78.0 | |

[a]Pro = promyelocytes; Myel = myelocytes; Mta = metamyelocytes; Band = band forms.

For other abbreviations see legend to **Table I**.

The reactivity of anti-NCA serum with PCA extract of CGL-cells,NCA, and CEA standards compared by double immunodiffusion showed identity or immunological relationship of the NCA extracted from leukaemic granulocytes and NCA earlier isolated from normal lung tissues (Figure 2). Additional absorption on an agarose plate of anti-NCA serum with PCA extracts of normal lung or spleen tissues removed the reactivity of this anti-serum not only with the extracts but also with CGL cells. Removing the activity with the the NCA standard was dependent on the concentration of normal tissue PCA extracts used in additional absorptions as an NCA source.

**Figure 2** Double immunodiffusion of anti-NCA serum with 1-NCA, 2-PCA-CGL, 3-CEA.

### Discussion

These results demonstrate that: (i) AML blasts lacking the ability to mature (M0 type) and mono-blasts did not contain NCA; (ii) individual AML blasts with features of maturation (M1, M2 types) and some blasts from myeloblastic crisis of CGL showed a limited expression of cytoplasmic NCA; (iii) the number of NCA containing cells increased as the more mature granulocyte fractions were isolated on Ficoll-Hypaque density-gradients; and (iv) myelocytic NCA is immunologically related if not identical with NCA isolated from lung tissue.

The stage in which NCA appears in the course of granulocyte differentiation is interesting mainly in relation to understanding the biological role of this glycoprotein. Our studies indicated that NCA appears early in the differentiation process of the myeloid cell series and is detectable in some differentiated myeloblasts (M1, M2 type leukaemias). These observations are concordant with earlier data of Burtin *et al.* (1979, 1980) suggesting that NCA can play a role in protection of granulocytic cells against their own enzymes. Other authors (Heumann *et al.*, 1979; Wahren *et al.*, 1979, 1980) were not able to detect NCA in myeloblasts and believed that it is a marker of more mature myeloid cells. Application of density-gradient centrifugation to separate granulocytes permitted the study of NCA distribution in

**Table III** NCA content in peripheral blood cells separated by density gradient centrifugation in CGL, CGL-BC patients and normal donors

| *Material* | *Number of cases* | *Density layer* ($g\,ml^{-1}$) | *Wright-Giemsa morphology (mean % and range)* Blasts Pro | Myel Mta | Band PMN | Eos[a] Bas | Lym | IF test $NCA^+$ *cells (mean % and range)* | *NCA serum (mean level in* $ng\,ml^{-1}$) |
|---|---|---|---|---|---|---|---|---|---|
| CGL | 9 | dextran | 6.7 (0–18) | 36.5 (23–71) | 50.0 (11–66) | 2.6 (0–10) | 4.2 (0–8) | 79.8 (42–90) | 142.0 |
| | | 1.06–1.07 | 3.6 (1–15) | 49.8 (34–59) | 37.4 (23–55) | 4.8 (0–8) | 4.4 (0–14) | 57.4 (44–74) | |
| | | 1.08–1.09 | 1.2 (0–4) | 25.0 (19–38) | 67.9 (55–81) | 5.2 (0–10) | 0.7 (0–4) | 92.5 (80–99) | |
| | | 1.105 | | 21.5 (16–28) | 70.3 (66–79) | 8.2 (0–15) | | 92.8 (91–96) | |
| CGL-BC | 5 | dextran | 22.3 (6–53) | 36.7 (14–63) | 41.0 (14–78) | | | 64.8 (57–84) | 100.0 |
| | | 1.05 | 62.0 (50–76) | 30.0 (12–40) | 8.0 (4–14) | | | 18.3 (7–40) | |
| | | 1.06–1.07 | 21.0 (11–33) | 38.3 (33–43) | 37.0 (29–48) | 3.0 (0–9) | 0.7 (0–2) | 53.6 (25–73) | |
| | | 1.08–1.09 | 9.0 (5–17) | 21.7 (3–49) | 65.3 (51–86) | 3.5 (0–17) | 0.5 (0–4) | 86.1 (60–98) | |
| | | 1.105 | 0.7 (0.2) | 14.6 (6–22) | 82.0 (74–94) | 2.7 (0–8) | | 83.7 (65–97) | |
| Normal granulocytes | 6 | 1.105 | | | 92.0 (79–99) | 3.0 (0–10) | 5.0 (0–21) | 90.0 (80–99) | 30.0 |

[a]Eos = Eosinophils; Bas = basophils.

For other abbreviations see legends to **Tables I and II**.

**Table IV** Percentage of $NCA^+$ cells in peripheral blood cells separated by density-gradient centrifugation in a patient with CGL-BC

| Density layer ($g ml^{-1}$) | *Wright-Giemsa morphology* (%) Blasts Pro | Myel Mta | Band PMN | Lym | *IF test* (%) $NCA^+$ Blasts | $NCA^+$ Myel | Mta $NCA^+$ Band | $NCA^+$ PMN |
|---|---|---|---|---|---|---|---|---|
| 1.05 | 33 | 51 | 16 | 0 | 7 | 46 | 0 | 0 |
| 1.06 | 22 | 46 | 32 | 0 | 14 | 58 | 64 | 78 |
| 1.07 | 11 | 39 | 48 | 2 | 22 | 72 | 64 | 70 |
| 1.08 | 2 | 16 | 82 | 0 | 0 | ND[a] | 75 | 72 |
| 1.09 | 0 | 14 | 86 | 0 | 0 | 0 | 83 | 98 |

[a]ND = not done.
For other abbreviations see legends to **Tables I** and **II**.

individual stages of granulocyte differentiation and comparison of IF results with phase-contrast appearances permitted an estimate of the number and type of NCA positively reacting cells in different fractions. It is worth emphasizing that independently of the increasing NCA content in morphologically more mature granulocytes, even myeloblasts and myelocytes focused in less dense Ficoll-Hypaque layers expressed a lower percentage of NCA positive cells than the same cellular forms from denser layers. It is probable that $NCA^+$ and $NCA^-$ myeloblasts and myelocytes could represent slight differences in maturation stage and density of these forms of granulocytes.

The mean NCA serum levels in CGLs were higher than in normal subjects and similar to those estimated by Frenoy *et al.* (1982). In our studies we noted large variations of serum NCA levels in individual cases. It is possible that values of circulating NCA in individual patients are dependent rather upon the number of NCA containing cells than on WBC count. AML patients with a small percentage of positively reacting cells had low serum NCA level, even when their WBC count was high. It could also be explained by the low rate of NCA synthesis in the immature leukaemic cells (Frenoy *et al.*, 1982). On the other hand, the ability to secrete NCA into the blood circulation from the myeloid cell series may play an important role in the regulation of the NCA serum level.

According to some authors (Bordes *et al.*, 1975; Heumann *et al.*, 1979; Nap *et al.*, 1983) myelocytic NCA is immunologically identical to NCA isolated from normal lungs. Our preliminary results showed an immunological relationship between lung and myelocytic NCA. However, detailed immunoprecipitation studies revealed some antigenic heterogeneity in NCA molecules since the precipitation lines with anti-NCA serum given by standard NCA and individual extracts of normal lung, spleen, or CGL cells, did not always show complete identity (unpublished data). In our opinion this interesting problem demands more detailed study, especially since recently Chavanel *et al.* (1983) revealed the existence of at least two epitopes on the specific moiety of NCA.

This work was supported by the Polish National Cancer Programme PR-6, Grant No. 2113. We thank Mrs Teresa Gruszczyńska for her excellent technical assistance.

### References

BENNET, J.M., CATOVSKY, D., DANIEL, M.T. & 4 others. (1976). Proposals for the classification of the acute leukaemias. *Br. J. Haematol.*, **33**, 451.

BORDES, M., KNOBEL, S. & MARTIN, F. (1975). Carcinoembryonic antigen (CEA) and related antigens in blood cells and hematopoietic tissues. *Eur. J. Cancer*, **11**, 783.

BURTIN, P., QUAN, P.C. & SABINE, M.C. (1975). Nonspecific cross-reacting antigen as a marker for human polymorphs, macrophages and monocytes. *Nature*, **255**, 714.

BURTIN, P., FLANDRIN, G. & FONDANECHE, M.C. (1979). Studies on the presence of NCA in human myeloid cells. In: *Carcino-Embryonic Proteins*. (Ed. Lehmann), Elsevier/North-Holland Biomedical Press, Vol. 1, p. 25.

BURTIN, P., FLANDRIN, G. & FONDANECHE, M.C. (1980). Presence of NCA (non-specific cross-reacting antigen) in the cells of the human granulocytic series. *Blood Cells*, **6**, 263.

CHAVANEL, G., FRENOY, N., ESCRIBANO, M.J. & BURTIN, P. (1983). Production of monoclonal antibodies against the non-specific cross-reacting antigen (NCA). Oncodevel. *Biol. Med.*, **4**, 209.

FRENOY, N., BEN-BUNANT, M., BERRUEL, C. & 5 others. (1982). An investigation of factors influencing serum NCA (non-specific cross-reacting antigen) level in patients with chronic myeloid leukaemia. *Br. J. Cancer*, **46**, 765.

GADLER, H., BREMME, K., WAHREN, B. & HAMMARSTRÖM, S. (1978). CEA and NCA in amniotic fluid of normal and abnormal pregnancies. *Cancer*, **42**, 1579.

HARŁOZIŃSKA, A., POTOMSKI, J., ŁAWIŃSKA, B., NOWOROLSKA, A. & RICHTER, R. (1982). High and low Fc IgG-receptor expression in human chronic granulocytic leukaemia cells. *Br. J. Cancer*, **45**, 194.

HARŁOZIŃSKA, A., RICHTER, R., ALBERT, Z. & ZAWADZKA, H. (1983). Antigenic heterogeneity of human lung cancers. *J. Natl Cancer Inst.*, **70**, 427.

HEUMANN, D., CANDARDJIS, Ph., CARREL, S. & MACH, J.-P. (1979). Identification of the normal glycoprotein (NGP) crossreacting with CEA as a differentiation antigen of myeloid cells and macrophages. In: *Carcino-Embryonic Proteins*. (Ed. Lehmann), Elsevier/North-Holland Biomedical Press, Vol. 2, p. 4.

IBRAHIM, A.N., ROBINSON, R.A., MARR, L., ABDELAL, A.T. & NAHMIAS, A.J. (1979). Tumor-associated antigens in cervical cancer tissues and in sera from patients with cervical cancer or with head and neck cancer. *J. Natl Cancer Inst.*, **63**, 319.

KROP-WATOREK, A., SEDLACZEK, P. & LISOWSKA, E. (1983). The subunit structure of non-specific cross-reacting antigen (NCA). *Mol. Immunol.*, **20**, 777.

KULA, J., HARŁOZIŃSKA, A., RICHTER, R., ALBERT, Z., SWARD, J. & GAWLIKOWSKI, W. (1983). Carcino-embryonic antigen in gynecologic cancers. Immuno-histochemical localization and serum levels. *Tumori*, **69**, 23.

MACH, J.-P. & PUSZTASZERI, G. (1972). Carcino-embryonic antigen (CEA): Demonstration of a partial identity between CEA and a normal glycoprotein. *Immunochemistry*. **9**, 1031.

NAP, M., TEN HOOR, K.A. & FLEUREN, G.-J. (1983). Cross-reactivity with normal antigens in commercial anti-CEA sera, used for immunohistology. The need for tissue controls and absorptions. *Am. J. Clin. Pathol.*, **79**, 25.

PATTENGALE, P.K., TAYLOR, C.R., PHILL, B.C., ENGVALL, E. & RUOSLAHTI, E. (1980). Direct tissue visualization of normal cross-reacting antigen in neoplastic granulocytes. *Am. J. Clin. Pathol.*, **73**, 351.

ROGERS, G.T. (1983). Carcinoembryonic antigen and related glycoproteins. Molecular aspects and specificity. *Biochim. Biophys. Acta*, **695**, 227.

VON KLEIST, S., CHAVANEL, G. & BURTIN, P. (1972). Identification of an antigen from normal human tissue that cross-reacts with the carcinoembryonic antigen. *Proc. Natl Acad. Sci.*, **69**, 2492.

WAHREN, B., HAMMARSTRÖM, S., GAHRTON, G. & HÖRNSTEN, P. (1979). Single cell quantification of NCA and CEA in myeloid cells. In: *Carcino-Embryonic Proteins*. (Ed. Lehmann), Elsevier/North-Holland Biomedical Press, Vol. 2, p. 15.

WAHREN, B., GAHRTON, G. & HAMMARSTRÖM, S. (1980). Nonspecific cross-reacting antigen in normal and leukemic myeloid cells and serum of leukemic patients. *Cancer Res.*, **40**, 2039.

ZAWADZKA, H., LISOWSKA, E., HARŁOZIŃSKA, A., ALBERT, Z., RICHTER, R. & JANUSZ, M. (1979). Differences in the purification effect of carcino-embryonic antigen (CEA) from the three different hepatic metastases of rectum carcinoma. *Neoplasma*, **26**, 157.